# PredHS: a web server for predicting protein–protein interaction hot spots by using structural neighborhood properties

**DOI:** 10.1093/nar/gku437

**Published:** 2014-05-22

**Authors:** Lei Deng, Qiangfeng Cliff Zhang, Zhigang Chen, Yang Meng, Jihong Guan, Shuigeng Zhou

**Affiliations:** 1 School of Software, Central South University, Changsha 410075, China; 2 Department of Computer Science and Technology, Tongji University, Shanghai 201804, China; 3 Department of Biochemistry and Molecular Biophysics and Center for Computational Biology and Bioinformatics, Columbia University, New York 10032, USAShanghai Key Lab of Intelligent Information Processing and School of Computer Science, Fudan University, Shanghai 200433, China; 4 Shanghai Key Lab of Intelligent Information Processing and School of Computer Science, Fudan University, Shanghai 200433, China

## Abstract

Identifying specific hot spot residues that contribute significantly to the affinity and specificity of protein interactions is a problem of the utmost importance. We present an interactive web server, PredHS, which is based on an effective structure-based hot spot prediction method. The PredHS prediction method integrates many novel structural and energetic features with two types of structural neighborhoods (Euclidian and Voronoi), and combines random forest and sequential backward elimination algorithms to select an optimal subset of features. PredHS achieved the highest performance identifying hot spots compared with other state-of-the-art methods, as benchmarked by using an independent experimentally verified dataset. The input to PredHS is protein structures in the PDB format with at least two chains that form interfaces. Users can visualize their predictions in an interactive 3D viewer and download the results as text files. PredHS is available at http://www.predhs.org.

## INTRODUCTION

Studies of molecular mechanisms for protein–protein interactions revealed that usually only a small subset of binding interfaces named hot spots account for the majority of binding free energy and are actually critical for stability and function of protein association (1). Identifying and understanding hot spots and their mechanisms on a large scale would have significant implications for practical applications including drug discovery (2) and protein design. Experimentally determined hot spots from alanine scanning mutagenesis experiments have been deposited in Alanine Scanning Energetics Database (ASEdb, (3)). Binding Interface Database (BID) presents experimentally verified hot spots at interfaces collected from literatures (4). However, the number of experimentally determined hot spots deposited in these databases is very limited since experimental techniques to identify hot spots are often labor intensive and expensive. Computational prediction of hot spots has become a practical alternative.

Current approaches for predicting hot spots can be classified roughly into three categories: (i) molecular dynamics (MD) simulations can simulate alanine substitutions and estimate the induced changes in binding free energy (ΔΔ*G*) at the atomic level. Some MD-based methods are successful to predict hot spots from protein interfaces (5–8); (ii) knowledge-based methods rely on empirically calibrated free energy functions, which include terms such as van der Waals and electrostatic interactions, hydrogen bonds and solvation energy, providing an alternative way to predict hot spots with much less computation. FOLDEF (9) and Robetta (10) belong to this group and were developed for the fast estimation of mutational free energy changes of a protein for hot spot identification; (iii) machine-learning methods, such as neural networks (11), decision trees (12), support vector machines (13–15), Bayesian networks (16,17), minimum cut trees (18) and random forests (19), have also been applied to detect hot spots in recent years. What's more, several hot spot databases, including HotRegion (20), HotSprint (21) and PCRPi-DB (17), were built based on computational methods.

Although substantial progress has been made, there is significant room for the improvement of protein hot spot prediction. For example, MD-based methods are not applicable for large-scale studies due to high computational cost. Knowledge-based methods are computationally much faster and reported results appear comparable to those from MD-based simulations (10). But the overall performance of these two groups of methods was inferior to machine-learning methods especially in the measure of recall (22). Machine-learning methods typically depend on the recognition of differences in features including physicochemical properties, evolutionary conservation and solvent accessible area. But specific biological properties for precisely identifying hot spots are often not fully exploited and the performance of the existing methods remains unsatisfactory. Moreover, the number of interacting hot spots of a protein is usually much smaller than the number of energetically unimportant interface residues. Existing methods usually have much higher specificity but rather lower recall since most classification algorithms tend to predict test samples as the majority class and may ignore the minority class when trained on the imbalanced data.

Recently, we developed an effective structure-based hot spot prediction method, PredHS (22), which integrates novel structural and energetic features with Euclidian and Voronoi neighborhoods in addition to conventionally used properties. Moreover, PredHS uses a two-step hybrid approach to select an optimal subset of features. Based on the selected features, a support vector machine (SVM) classifier and an ensemble model are built for prediction. We have benchmarked PredHS using a set of experimentally verified hot spot residues and an independent dataset. Results show that PredHS significantly outperforms the state-of-the-art methods and indicate that structural neighborhood properties are important determinants of hot spots (22).

Here, we present the PredHS web server, which is an automatized online implementation of the PredHS method. The server allows users to request new predictions for input PDB IDs or structures files provided in PDB format. The resulting predictions can be visualized in an interactive 3D viewer and downloaded as text files.

## METHODS

**Figure 1.**
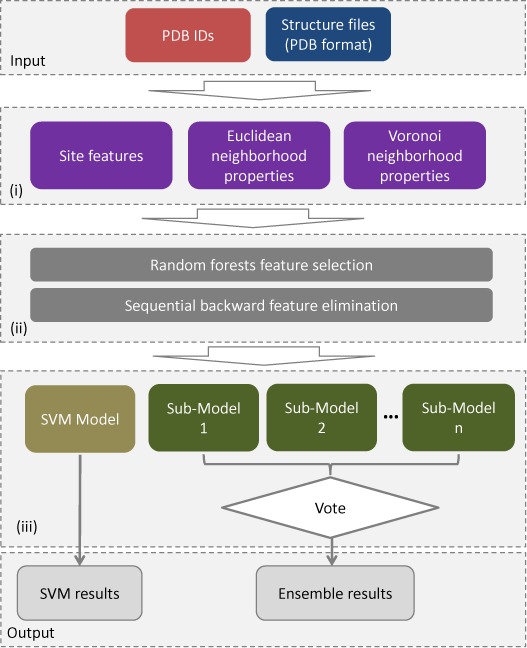
Flow chart of the PredHS web server. Input to the PredHS server can be protein structure files in PDB format or a list of PDB codes. After job submission, the server invokes three main component processes: (i) feature extraction: a set of 108 site features, 108 Euclidean neighborhood features and 108 Voronoi neighborhood features are extracted; (ii) feature selection: in the training process, a subset of 38 optimal features are selected by using a random forest algorithm and a sequential backward elimination method, these selected features are used for prediction and (iii) prediction models: PredHS-SVM and PredHS-Ensemble, where PredHS-Ensemble is an ensemble of *n* sub-models using a bootstrap resampling method to generate subsets. Finally, two groups of results are obtained (SVM results and Ensemble results), corresponding to the two predictors.

The computational approach used by the PredHS web server consists of three main component processes (Figure 1): (i) feature extraction: to extract a wide variety of sequence, structural and energy features, together with two types of structural neighborhoods; (ii) feature selection: a two-step feature selection process that combines random forest and a sequential backward elimination and (iii) predictor construction: two predictors (PredHS-SVM and PredHS-Ensemble) are built for identifying hot spots based on the optimally selected features.

### Structural neighborhood properties

A total of 108 features are extracted to describe potential hot spot residues. In addition to conventionally used properties, many novel structural and energetic features are also used, including local structural entropy (23), side chain energy score (24), four-body pseudo-potential (25) and topographical score (16,26). Based on these features, we propose a new way to calculate two types of structural neighborhood properties using Euclidean distance and Voronoi diagram.The Euclidean property of a target residue is defined by summing up the values of the properties in the neighborhood. The Euclidean neighborhood is a group of residues located within a sphere of 5 Å defined by the minimum Euclidean distances between each heavy atom of the surrounding residues and each heavy atom of the target residue. The Voronoi diagram (27) is another way to calculate structural neighborhood properties, which partitions a 3D space (a protein structure) into several Voronoi regions, each of which contains a point (heavy atom of a residue). A pair of residues are said to be each other's neighbor when there is at least one pair of their heavy atoms has a Voronoi facet in common. The Voronoi partition is computed by Qhull (28). This definition is based on geometric partitioning rather than the use of an absolute distance cutoff, and hence is considered to be more robust (29).

### Two-step feature selection

To remove potentially redundant ones from the whole set 108 features we implement a two-step strategy. The first step is to evaluate the importance of each candidate feature by the mean decrease Gini index (MDGI) with the random forest (RF) package in R (30). A higher MDGI score means the feature is more informative for classifying an interface residue into hot spots and non-hot spots. In PredHS, 77 features with MDGI Z-score larger than 2.5 are selected. In the second step, redundant features are removed by sequential backward elimination (SBE) with 10-fold cross-validation. The SBE algorithm sequentially removes features from the whole feature set till an optimal feature subset is obtained. A feature is removed if its removal maximizes the performance of the predictor. Finally, an optimal set of 38 features is obtained for building prediction models (22).

### Prediction models

Two classifiers were built for hot spot prediction: one is PredHS-SVM, which is implemented with LIBSVM package (31) using radial basis function (RBF) as the kernel; the other is an ensemble classifier, PredHS-Ensemble, which is built to handle the problem of imbalance in classification. PredHS-Ensemble uses an ensemble of *n* sub-models that employ an asymmetric bootstrap resampling approach to generate subsets. Each subset contains all of the hot spots and a subset of non-hot spots that is generated using random bootstrap sampling and has the same size as hot spots. The final results are calculated by majority votes among the outputs of the *n* sub-models.

## WEB SERVER INTERFACE

Users can upload a file of a protein structure in the PDB format or simply input a PDB code to start a job. The input structure should contain at least two chain identifiers forming an interface. Multiple structures can be submitted in one run. Users could choose to leave their email address or a job title to conveniently retrieve the results. The PredHS server first checks the validity of the input structure, and once confirmed, it progresses to the second step for users to select the query protein and its partners. When the selection is done, users can submit the prediction job by clicking the ‘Submit’ button.

**Figure 2.**
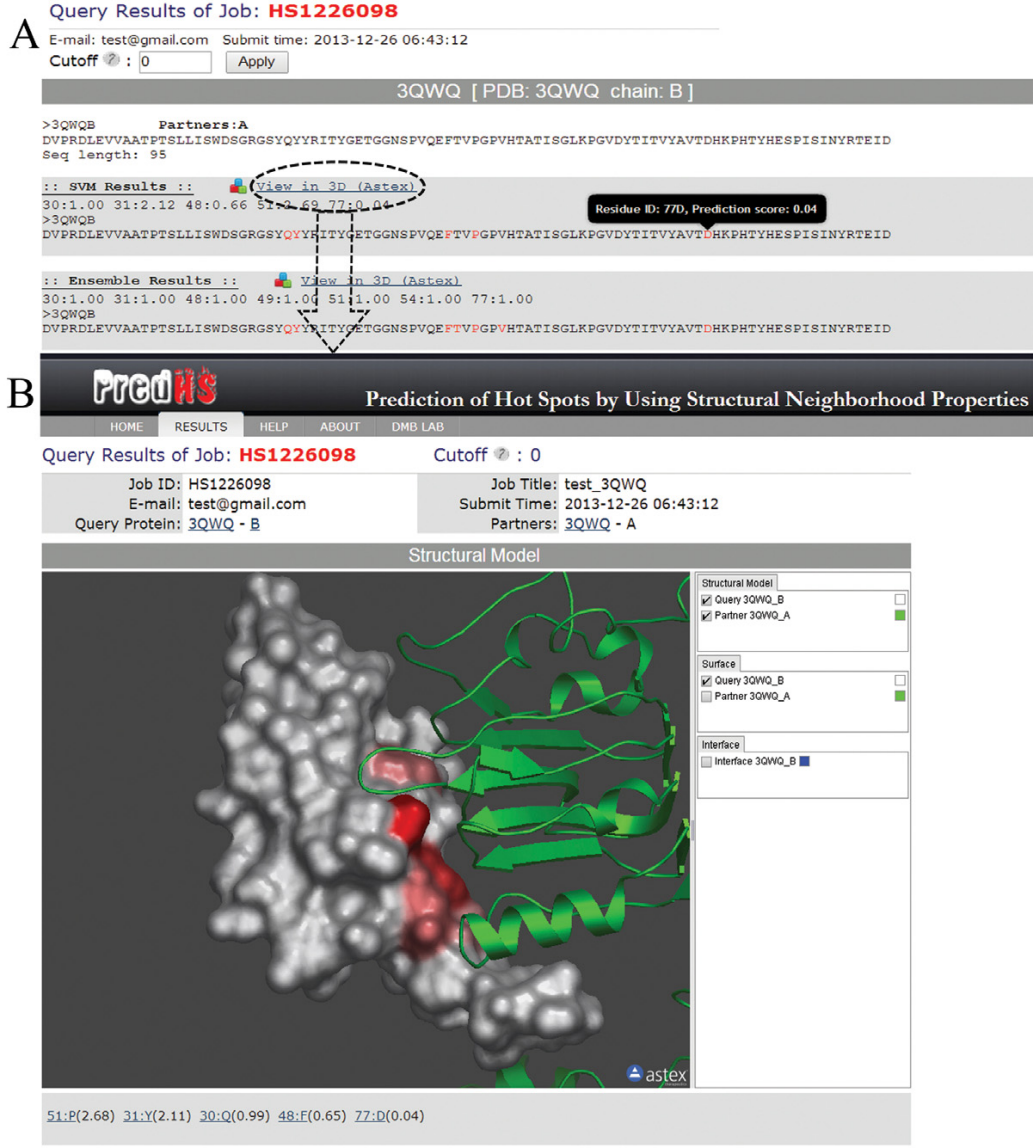
A snapshot of PredHS prediction output. (**A**) SVM results and Ensemble results of a job are listed. Predicted hot spots are colored in red. By default, PredHS predicts interfaces to be hot spots with a default cutoff 0 which is adjustable by the user. Users can put the mouse over a residue to view its residue ID and predicted score. (**B**) Interactive 3D view for a prediction. Predicted hot spots are colored according to their predicted scores. Residues with score higher than zero are shown from light red to red as the score increases.

A typical query takes no more than 30 min to run. For each submitted structure, the server returns two lists of residues and their associated scores to be hot spot, corresponding to PredHS-SVM and PredHS-Ensemble, respectively (Figure 2A). The red residues in the query sequence are predicted hot spots. Users can view the residue ID and its associated score by putting the mouse over the residue. The higher the score is, the more likely a given residue is a hot spot. The results can be downloaded in text or visualized in an interactive 3D viewer AstexViewer (32) by following the ‘View in 3D’ link. As shown in Figure 2B, predicted hot spots are colored according to their associated scores.

## RESULTS

The PredHS web server trains prediction models based on a dataset of 265 experimentally mutated interface residues obtained from ASEdb (3) and the published data of Kortemme and Baker (10), among which 65 are hot spots. To make a fair comparison with other methods, we use an independent test dataset extracted from the BID database that contains alanine-mutation experiments of a different set of 127 interface residues, of which 39 are identified as hot spots.

We calculated a variety of measures to evaluate the predictions:

\begin{equation*} {\rm Recall} = {\rm TP}/({\rm TP} + {\rm FN}); \end{equation*}

\begin{equation*} {\rm Specificity} = {\rm TN}/({\rm FP} + {\rm TN}); \end{equation*}

\begin{equation*} {\rm Precision }= {\rm TP}/({\rm TP }+ {\rm FP}); \end{equation*}

\begin{equation*} {\rm Accuracy }= ({\rm TP }+ {\rm TN})/({\rm TP }+ {\rm FP }+ {\rm TN }+ {\rm FN}); \end{equation*}

\begin{equation*} {\rm CC} = \frac{{({\rm TP}{\times}{\rm TN }- {\rm FP}{\times}{\rm FN})}}{{\sqrt {({\rm TP }+ {\rm FN})({\rm TP }+ {\rm FP})({\rm TN }+ {\rm FP})({\rm TN }+ {\rm FN})} }}; \end{equation*}

\begin{equation*} F1 = 2{\times}{\rm Precision}{\times}{\rm Recall}/({\rm Precision} + {\rm Recall}). \end{equation*}

Here TP, FP, TN and FN are true positive, false positive, true negative and false negative counts. We also calculated the area under the receiver operating characteristic (ROC) curve (AUC).

**Figure 3.**
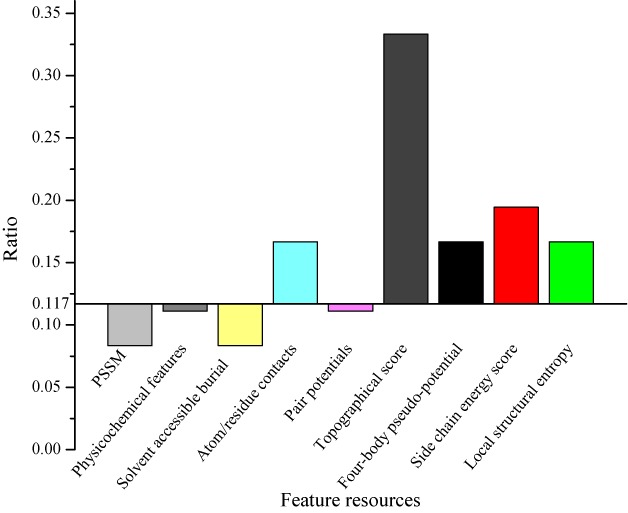
Relative contribution of different feature resources. The y-axes show the ratio of the individual feature resource occurred in the selected optimal features. The reference ratio of 0.117 is the ratio of 38 selected features out of the total number of features.

PredHS predicts hot spots using an optimal feature set of 38 features, which are selected from the combination of 108 site features, 108 Euclidean features and 108 Voronoi features with the proposed two-step feature selection approach. These features are calculated based on heterogeneous information resources, including position-specific scoring matrix (PSSM) (33), physicochemical features (34), solvent accessible burial (35), atom/residue contacts (13), pair potentials (36), topographical score (16,26), four-body pseudo-potential (25), side chain energy score (24) and local structural entropy (23). To analyze the relative contribution of different feature resources, we calculated the ratio of each feature resource occurred in the selected optimal set (37). The ratio of 0.117 is used as a reference ratio since it is the ratio of selected 38 optimal features out of the total number of features. From Figure 3, we can see that the topographical score contributes most to the hot spot identification, followed by the side chain energy score, atom/residue contacts, four-body pseudo-potential and local structural entropy.

We used 10-fold cross validation on the training dataset to evaluate the predictive power of structural neighborhood properties and selected optimal features. Five SVM classifiers were built and tested using five groups of features, including site, sequence, Euclidean, Voronoi and optimal features. The sequence features are generated with a sliding window of 21, which includes 10 residues upstream and 10 residues downstream of the target residue in the protein sequence. As shown in Table 1, classifiers with structural neighborhood properties (Euclidean and Voronoi) achieve better performance than those using site and sequence features in terms of AUC score. The classifier with linear sequence neighborhood properties is significantly worse than the others, and thus the sequence features are not included in the combination. PredHS-SVM with the optimal features achieves the best performance, suggesting that the proposed two-step feature selection algorithm can effectively improve the prediction.

**Table 1.** Prediction performance comparison of classifiers with different types of features (site, sequence, Euclidean, Voronoi and optimal subset)

Feature types	AUC	Accuracy	Recall	Specificity	Precision	CC	*F*1
Site	0.81	0.82	0.62	0.89	0.66	0.52	0.63
Sequence	0.76	0.80	0.39	0.93	0.77	0.42	0.46
Euclidean	0.82	0.82	0.57	0.91	0.67	0.50	0.60
Voronoi	0.83	0.84	0.60	0.92	0.75	0.57	0.65
Optimal subset (PredHS-SVM)	0.87	0.88	0.75	0.93	0.79	0.69	0.76

Furthermore, we compared PredHS with other five state-of-the-art methods, including Robetta (10), FOLDEF (9), HotPoint (38), KFC2a and KFC2b (15). Each method has a companion web server or a stand-alone software. Results of the independent BID dataset are shown in Table 2. PredHS significantly outperforms the existing methods in the five performance measures (accuracy, specificity, precision, CC and *F*1 score). Although KFC2a has a similar recall value (0.74) to that of PredHS-Ensemble, the specificity (0.74) and precision (0.56) of KFC2a are much lower than that (0.80 and 0.63) of PredHS-Ensemble.

**Table 2.** Prediction performance comparison on the independent BID dataset. Maximum value(s) of each performance measure is(are) highlighted in bold.

Methods	Accuracy	Recall	Specificity	Precision	CC	*F*1
Robetta	0.70	0.33	0.86	0.52	0.23	0.41
FOLDEF	0.68	0.26	0.87	0.48	0.16	0.33
HotPoint	0.69	0.59	0.74	0.5	0.31	0.54
KFC2a	0.74	**0.74**	0.74	0.56	0.41	0.64
KFC2b	0.79	0.59	0.87	0.68	0.47	0.63
PredHS-SVM	**0.83**	0.59	**0.93**	**0.79**	**0.57**	**0.68**
PredHS-Ensemble	0.79	**0.74**	0.80	0.63	0.53	**0.68**

## CONCLUSION

The PredHS web server provides an automated platform to predict hot spots from interfaces. In contrast to the approaches based on the recognition of differences in physicochemical properties, evolutionary conservation and solvent accessible area, an advantage of PredHS is that it integrates Euclidian and Voronoi neighborhoods together with a variety of heterogeneous information, including sequence-based, structure-based and energetic features. What's more, PredHS uses a two-step feature selection approach, providing an effective way for selecting an optimal subset of features within a reasonable computational cost, which improves the prediction performance and reduces the risk of over-fitting.

A limitation of PredHS and many other hot spot prediction methods is that they can only identify hot spots from known protein interfaces, which means that the input to these methods should be protein complexes forming interfaces other than monomers. We plan to improve PredHS by using structural alignment methods to detect hot spots from predicted interfaces, and thus make monomer input possible.

PredHS has been in service for >10 months and it is under continuous improvement. We hope PredHS can be applied to a wide range of hot spot identification and further functional analysis and so to provide a practical tool for biologists.
